# Acute Ischemic Stroke in an Eight-Year-Old Male With Elevated Factor VIII Activity and SARS-CoV-2 Antibodies

**DOI:** 10.7759/cureus.24982

**Published:** 2022-05-13

**Authors:** Seth J Deskins, Matthew Mamone, Samuel Luketich, Arin Jennings, Sydney Downey, Jacob Gelman, Richard Brant, Collin John

**Affiliations:** 1 Internal Medicine/Pediatrics, West Virginia University School of Medicine, Morgantown, USA; 2 Pediatrics, West Virginia University School of Medicine, Morgantown, USA

**Keywords:** vascular neurology, pediatrics medicine, factor viii, sars-corona virus 2, stroke

## Abstract

Acute ischemic stroke (AIS) is a significant source of morbidity and mortality and is one of the top causes of death in the United States. Of these patients, most are elderly individuals, compared to a limited proportion of cases seen in pediatrics. AIS is classically associated with age-dependent atherosclerotic disease processes secondary to comorbidities such as diabetes and hypertension. When considering the pediatric population, stroke is far less common and often requires workup of other underlying etiologies that create a hypercoagulable state. Here we present a case of an eight-year-old male with a left middle cerebral artery (MCA) ischemic stroke in the setting of increased factor VIII activity and SARS-CoV-2 antibodies.

## Introduction

More than three-fourths of ischemic strokes occur in people over the age of sixty-five [1]. Albeit less common in pediatrics compared to adults, stroke still remains a significant contributor to morbidity and mortality in this population. Underlying hypercoagulable states, arrhythmias, or embolic events should be considered in pediatric patients presenting with acute ischemic stroke. A broad workup should be pursued to determine the exact etiology. Treatment can be difficult given the lack of large studies with standard treatments such as administration of tissue plasminogen activator (tPA). Here we present a case of acute stroke in an otherwise healthy eight-year-old male with a multifactorial prothrombotic etiology.

## Case presentation

An eight-year-old Caucasian male with a body mass index of 21.6 and a past medical history of attention deficit hyperactivity disorder on methylphenidate and a cystic fibrosis carrier presented as direct admission to our children's hospital. He had a pertinent family history of an uncle with a fatal myocardial infarction in his 30s of unclear etiology. Four weeks prior to the presentation, he endorsed headache, malaise, and fever. He was evaluated at local urgent care, where he was febrile at 39.4°C. He was diagnosed with pneumonia and subsequently started on amoxicillin. Rapid respiratory viral antigen testing, which included SARS-CoV-2, was negative. Of note, the patient had not been vaccinated for SARS-CoV-2 prior to illness and subsequent hospitalization. He failed to have clinical improvement and presented after eleven days again to his primary care provider's office. There, he was given azithromycin and had subjective improvement in symptoms. Two weeks after the onset of symptoms, his mother noted that he had right-sided facial droop and difficulty dressing himself secondary to right arm weakness. He presented to an outside emergency department, where he underwent immediate computed tomography (CT) imaging, which revealed no acute intracranial process before being transferred for admission to our tertiary care center. Electrocardiogram (EKG) performed there showed a normal sinus rhythm, and basic laboratory workup was unremarkable. Per outside records, tissue plasminogen activator (tPA) was not considered likely due to reports of waxing and waning of neurologic deficits, making a true last known normal difficult to ascertain along with lack of pediatric neurology expertise at the facility. 

**Figure 1:**
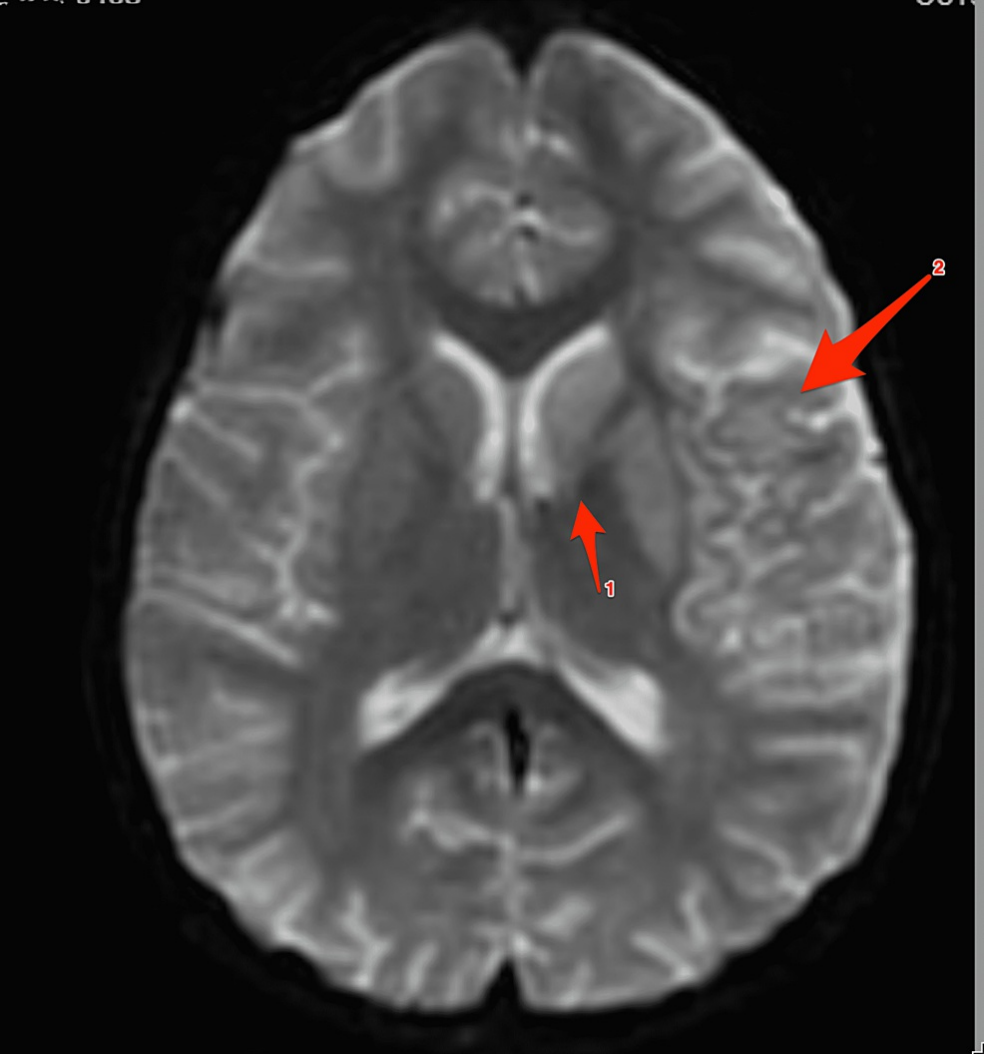
MRI of the brain obtained upon presentation to an outside facility Arrow 1: diffusion restriction at the left caudate nucleus. Arrow 2: diffusion restriction in the left parietal and temporal lobes.

Following initial imaging, magnetic resonance imaging of the brain with angiography revealed an infarct of the cortex in the territory of the left middle cerebral artery (MCA) that involved a large portion of the left parietal lobe and small volumes of the left frontal and temporal lobes. Additionally, acute infarction was seen involving the left basal ganglia, left thalamus, and left midbrain (Figure 1). No large vessel occlusions (LVO) were noted, making the possibility of intervention with thrombectomy not possible. Routine laboratory testing, including a complete blood count and basic metabolic panel, was unremarkable aside from mild thrombocytosis (536 K/mm^3^; normal: 150-450 K/mm^3^). Hepatic function panel revealed hypoalbuminemia (2.9 gm/dL; normal: 3.5-5.0 gm/dL) and a modestly elevated aspartate aminotransferase (46 units/L; normal: 10-42 units/L). His erythrocyte sedimentation rate was elevated at 22 mm/hr (normal: 0-10 mm/hr). Other tests, including C-reactive protein, antinuclear antibody, Rickettsia assay, Lyme assay, and urine drug screen, were unremarkable.

**Figure 2:**
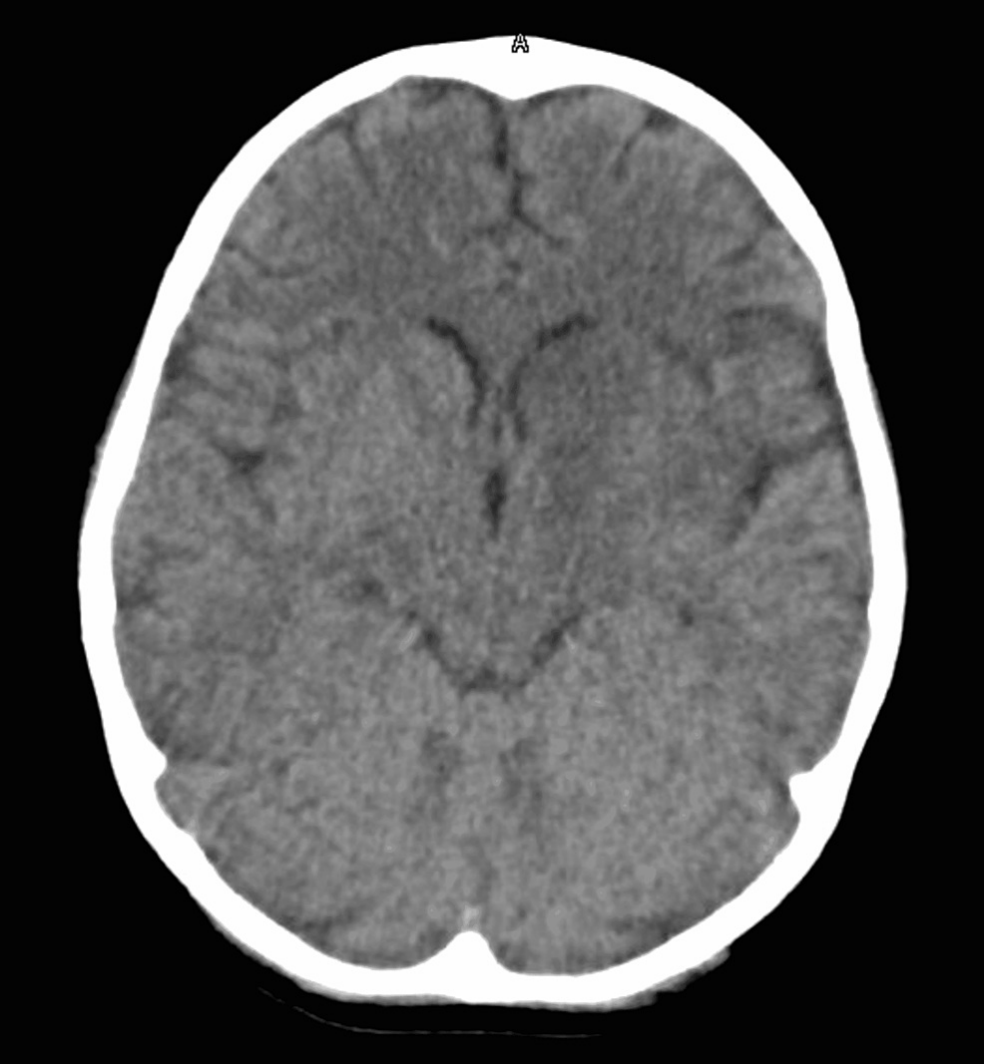
CT of the head obtained 72 hours after stroke No evidence of hemorrhagic conversion was noticed.

Further workup was pursued to determine the etiology of this child's acute stroke. Venous duplexes of the upper and lower extremities and a transthoracic echocardiogram with a shunt study showed no thromboembolic sources. An electrocardiogram showed normal sinus rhythm, and telemetry was unrevealing for arrhythmias throughout his hospitalization. A hypercoagulable workup was completed, which included antithrombin III activity, lupus anticoagulant, protein C activity, protein S antigen, factor V and two gene mutations, factor VIII activity, and testing for SARS-CoV-2 antibodies. This was remarkable for elevated factor VIII activity (>300%; normal: 50-150%) and the presence of IgG SARS-CoV-2 antibodies, indicating prior infection as he was never vaccinated for SARS-CoV-2, even though eligible at the time with recent guideline changes in pediatrics. Pediatric neurology was consulted for additional recommendations. He was started on aspirin 81 mg daily, and statin therapy was considered but was not initiated. Seizure prophylaxis was initiated with levetiracetam 250 mg twice daily and planned to continue for a minimum of two weeks. A follow-up CT venogram at 72 hours post-stroke was stable without hemorrhagic conversion or additional pathology (Figure 2). Motor weakness improved gradually during hospitalization, and he was discharged home with outpatient physical and occupational therapy. 

## Discussion

While uncommon in the pediatric population, stroke should not be prematurely dismissed in a patient presenting with concerning symptoms. However, there are some important stroke mimics to consider, including but not limited to seizure, intracranial neoplasm or abscess, drug toxicity, and idiopathic intracranial hypertension [2]. Only after careful consideration of stroke and other more serious conditions should providers shift focus to benign etiologies like migraine, musculoskeletal abnormalities, or conversion disorder.

Given this patient's age, it is important to evaluate the etiology underlying his ischemic event. The coagulation cascade is a complex physiological process with numerous components, and thus there are many opportunities for pathologic change to occur, increasing the risk of thrombosis or bleeding. Studies suggest that up to 13% of pediatric ischemic strokes are attributed to an underlying prothrombotic state, a rate much higher than in adults. Compounding this, approximately a quarter of pediatric patients found to be hypercoagulable have more than one identifiable cause [3]. Hypercoagulability associated risk factors to consider are factor V Leiden, protein C and S deficiencies, plasminogen and antithrombin III deficiencies, elevated factor VIII levels/activity, sticky platelet syndrome, dysfibrinogenemia, tissue plasminogen activator deficiency, tissue plasminogen activator inhibitor pathologies, antiphospholipid antibodies, homocystinuria, and, more recently, recent/concurrent SARS-CoV-2 infections [4-5].

Increased factor VIII activity has been associated with increased myocardial infarctions and ischemic strokes. This increased activity is thought to increase thromboembolism by increasing thrombin generation [6]. Our patient had increased factor VIII activity, suggesting a potentially prothrombotic state. In addition to increased activity, increased factor VIII level is an independent risk factor for venous thromboembolism. Individuals with factor VIII levels greater than 1,500 IU/I are six times more likely to develop venous thromboembolism compared to those with levels less than 1,000 IU/I. It is thought that approximately 11% of the population has increased factor VIII levels, attributed to both environmental and genetic factors. Chronic inflammatory states such as obesity, hypertriglyceridemia, and diabetes can lead to increased levels, as increased age and recent surgery [7].

While our patient did not demonstrate any of those chronic inflammatory states, he did have evidence of recent SARS-CoV-2 infection, which creates a proinflammatory state and potentially contributed. SARS-CoV-2 infections have now been well recognized to increase the risks of thrombosis. This is a more unique characteristic of SARS-CoV-2, as rates of thromboembolism have been significantly higher in these infected patients compared to patients infected with other bacteria or viruses. Thrombus formation is more likely to occur in patients requiring ICU-level care, but it is possible in individuals with less severe infections as well [8]. Furthermore, there have been cases of cerebral vascular accidents reported after infection, as seen in our patient. Our patient's recent vague respiratory illness likely was secondary to a SARS-CoV-2 infection, given his positive antibody titers. His negative test was likely a function of a viral load not sufficient enough to trigger a positive test. It is hypothesized that SARS-CoV-2 leads to hypercoagulability by triggering a profound proinflammatory immune response leading to endothelial injury within vessels and subsequently triggering the coagulation cascade [9]. Combined with elevated factor VIII activity, recent SARS-CoV-2 infection likely created a hypercoagulable state, contributing to his ischemic stroke. 

Another potential source of stroke is an embolus. An embolus originates in one location and travels elsewhere via the vasculature to occlude a vessel. The vast majority of emboli are cardioembolic. Atrial fibrillation, among other arrhythmias, is a risk factor for stroke. The well-known scoring system CHADsVASC is used to estimate the risk of stroke in adults with atrial fibrillation. Lone atrial fibrillation, or atrial fibrillation in the absence of structural heart disease, is estimated to affect 7.5 per 100,000 children [10] and thus should always be considered. 

AIS has limited options for intervention in pediatrics. Airway management, intravenous access, and monitoring vitals and mental status should be the predominant initial focus [11]. The Food and Drug Administration has not approved tissue plasminogen activator (tPA) for pediatric usage outside of clinical trials, and such trials are difficult to initiate as the incidence of AIS is sparse at best in single centers. One retrospective study, which reviewed cases from 2000 to 2003, found that out of 9,257 children admitted for AIS, only 1.6% received thrombolytic treatment. Of note, the children who were administered tPA had a higher incidence of death compared to those who did not receive a thrombolytic intervention [12]. It is difficult to ascertain whether this was related to the severity of the ischemic insult or secondary to thrombolytic administration. A previous literature review analyzed 17 case reports of children who have received IV fibrinolysis, and outcomes include a 94% survival rate and 71% positive results of the intervention [13]. Therefore, the true benefits of thrombolysis remain unclear in children. Efforts to gain insight into the development of a pediatric thrombolytic dosage have also been made in the past; however, the trial was terminated prematurely due to a lack of enrollment [3]. Not surprisingly, the literature surrounding pediatric endovascular thrombectomy is even more sparse. 

Pediatric ischemic stroke creates a difficult clinical scenario for physicians, especially when symptoms fall within the standard thrombolytic window used for adult patients. Some have hypothesized that efforts to limit glutamate-induced excitotoxicity through the usage of N-methyl D-aspartate (NDMA) receptor antagonists, such as memantine, may be of utility in the treatment of AIS [14]. Unfortunately, no trials have studied clinical applicability in adults, let alone pediatric patients. Given a lack of other treatment options and the potentially devastating consequences of AIS, further investigation may be warranted. Conventional long-term treatment post-stroke mirrors adult strategies with aspirin and consideration of a statin if indicated [15], along with risk factor modification.

Post-stroke, the focus shifts to determining the underlying etiology and modification of risk factors such as hypertension, diabetes mellitus, and hyperlipidemia, if possible [3]. Treatment with aspirin, clopidogrel, or dual antiplatelet therapy remains controversial in pediatric patients [16]. Following pediatric AIS, the development of seizures is a concern. One study found that 59% of pediatric, post-stroke patients went on to have a seizure shortly thereafter, and a startling 41% of patients went on to develop epilepsy [17]. Due to this, seizure prophylaxis is typically started, at least in the acute setting.

## Conclusions

Ischemic stroke is an infrequent occurrence in the pediatric population, but when cases occur, extensive workup should be performed to identify the underlying cause. Here we present a case of a left MCA stroke in an eight-year-old male. Workup revealed elevated factor VIII activity and positive IgG SARS-CoV-2 antibodies, possibly together creating a hypercoagulable state leading to arterial thrombosis. This case is a reminder that the pediatric patient can be affected by ischemic stroke, and etiology, along with subsequent workup and management, can be quite different than adult counterparts. 
